# Splicy: a web-based tool for the prediction of possible alternative splicing events from Affymetrix probeset data

**DOI:** 10.1186/1471-2105-8-S1-S17

**Published:** 2007-03-08

**Authors:** Davide Rambaldi, Barbara Felice, Viviane Praz, Philip Bucher, Davide Cittaro, Alessandro Guffanti

**Affiliations:** 1 The IFOM-IEO Campus, Via Adamello, 16 – 20139 Milano, Italy; 2 ISREC, Ch. des Boveresses 155, Epalinges, Switzerland; 3 CNR-ITB, Via Fantoli 16/15 – 20138 Milano, Italy

## Abstract

**Background:**

The Affymetrix™ technology is nowadays a well-established method for the analysis of gene expression profiles in cancer research studies. However, changes in gene expression levels are not the only way to link genes and disease. The existence of gene isoforms specifically linked with cancer or apoptosis is increasingly found in literature. Hence it is of great interest to associate the results of a gene expression study with updated evidences on the transcript structure and its possible variants.

**Results:**

We present here a web-based software tool, Splicy, whose primary task is to retrieve data on the mapping of Affymetrix™ probes to single exons of gene transcripts and displaying graphically this information projected on the gene physical structure.

Starting from a list of Affymetrix™ probesets the program produces a series of graphical displays, each relative to a transcript associated with the gene targeted by a given probe. The information on the transcript-by-transcript and exon-by-exon mapping of probe pairs can be retrieved both graphically and in the form of tab-separated files. The mapping of single probes to NCBI RefSeq or EMBL cDNAs is handled by the ISREC mapping tables used in the CleanEx Expression Reference Database Project. We currently maintain these mappings for most popular human and mouse Affymetrix™  chips, and Splicy can be queried for matches with human and mouse NCBI RefSeq or EMBL cDNAs.

**Conclusion:**

Splicy generates probeset annotations and images describing the relation between the single probes and intron/exon structure of the target transcript in all its known variants. We think that Splicy will be useful for giving to the researcher a clearer picture of the possible transcript variants linked with a given gene and an additional view on the interpretation of microarray experiment data. Splicy is publicly available and has been realized in the framework of a bioinformatics grant from the Italian Cancer Research Association.

## Background

Alternative splicing is a biological process that generates multiple different transcripts from the same precursor mRNA. It is an important regulatory mechanism for eukaryotic gene expression occurring in about 40–60% of human genes.

The process is known to play critical roles in the regulation of development, cellular differentiation, maintenance of the differentiated state and apoptosis [2,3]. In addition, disruption of splicing is frequently associated with human diseases [4,5]. It is well known that cancer is strictly associated with relevant changes in gene expression; hence, it is crucial to know whether cancer involves sensible changes in mRNA splicing patterns and isoform expression levels.

Genome wide methods are providing a better and more complete understanding of the functional relevance of splice variants and genetic mechanisms of disease. Microarrays are now a widely used high-throughput technology for studying gene expression and regulation on a global scale.

Microarray technology, in particular the recently developed customized oligonucleotide microarrays, that include probes for the exon bodies and junctions is a key tool to detect alternative splicing [6]. The Affymetrix™ technology [7] is nowadays a well-established method for the detection of gene expression profiles. The Affymetrix™ DNA chip technology is based on hybridization of labeled RNA probes with gene-specific oligonucleotides. By detecting the intensity of hybridizing probes on the chip, the researcher can analyze the expression level of thousands of genes simultaneously. Since protocols used for microarray experiments tend to be biased towards the end of the gene, each gene is measured by a number of pairs of oligonucleotide probes spanning the 3' region of each mRNA. Whereas for alternative splicing it is important to have probes throughout all regions of the gene and at exon-exon junction [8]. So even though the Affymetrix™ technology for detection of changes in gene expression levels is not comparable to an "exon chip", we built a computational pipeline to extract information on changes in the transcript structure from Affymetrix™ gene expression data.

We have developed a software tool called Splicy for the detection and graphical representation of the location of Affymetrix™ probes on the human and mouse transcriptomes (RefSeq and mRNA transcripts), with a classification between probes that match a single exon and probes that match an exon junction ('junction probes'). Using this software it is possible to identify probesets lists matching different transcripts that correspond to the same gene locus. It is possible to predict from these probesets which probes could hybridize with exons that are skipped in some isoforms of a given gene, generating so-called 'splice diagnostic probesets'.

## Conclusion

**Figure 1:**
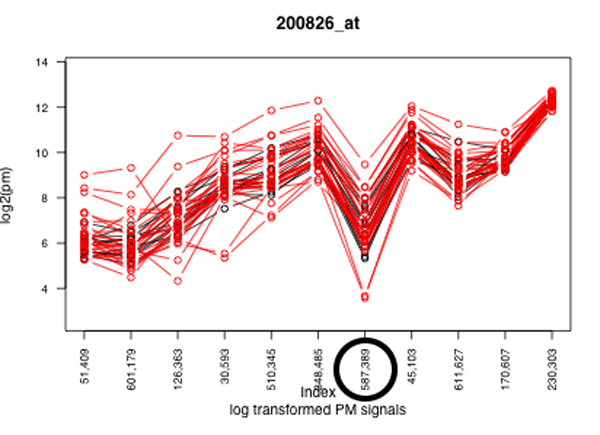
Bioconductor plot of Perfect Match signal for normal Breast tissue samples (black lines) and Breast cancer samples (red lines).

**Figure 2:**
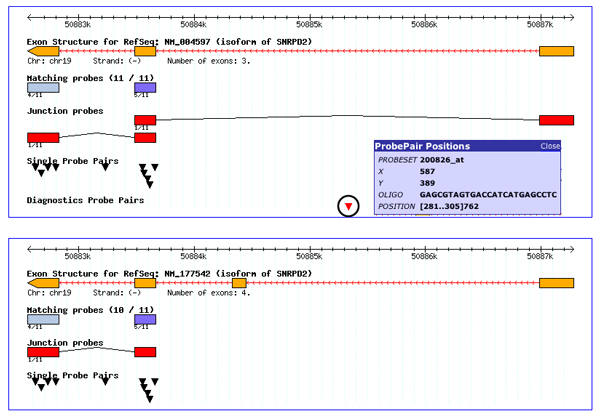
Splicy graphical report for Probeset 200826_at (GeneChip^® ^Human Genome U133A) and mapping on the two transcript variants for Gene SNRPD2. The probe at position X = 587 and Y = 389 corresponds to the probe pair with low signal intensity at Figure 2. This probe matches only with the variant of SNRPD2 that skips an exon, while the other variant doesn't maintain the same exon junctions.

Splicy is a web-based tool whose aim is to generate probeset annotations and images describing the relation between the single probes and intron/exon structure of the target gene. Using Splicy it is possible to highlight the differences in hybridization at the isoform level of the Probeset. Through a Bioconductor [9,10] function, we have made some plots of single probes Perfect Matches (PM) from hybridization samples of breast cancer and normal breast tissue. The single probe is labeled with X and Y position over the GeneChip^® ^array and the logarithm of the signal intensity is plotted on the Y-axis. Comparing these plots with the Splicy graphical display, we can observe that in some cases the single probe with the lowest signal is also the probe that hybridize with an exon that can be skipped in one of the isoforms of the gene. As an example: the PM plot for Probeset *200826_at *of GeneChip^® ^HG_U133A (Figure 1) can be compared with the corresponding Splicy output (Figure 2): the probe pair with the lowest signal in the plot matches with the junction between two exons on the Splicy output. If one of the two exons is skipped, a perfect hybridization of this probe pair is no longer possible, suggesting that the transcript variant 2 of the gene SNRPD2 is more likely to be present in the hybridized samples.

## Methods

### Architecture

**Figure 3:**
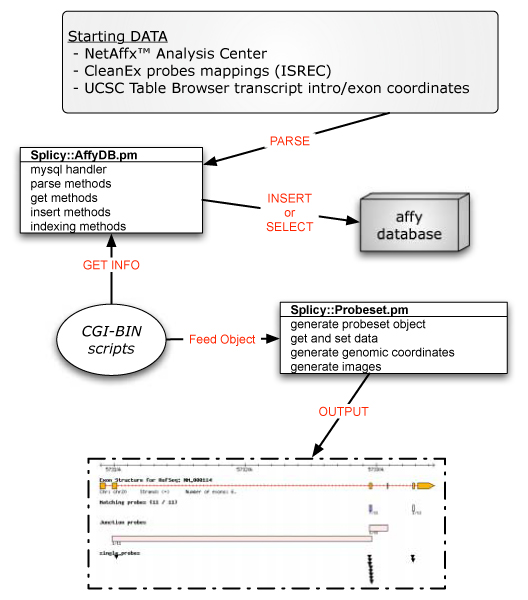
The server integrates annotation from different sources. A first module: Splicy::AffyDB is used to store data into a MySQL database, a second Perl Module handles this information during runtime and generates graphical reports. The two modules are called from a Perl CGI script.

We mantain a server (Figure 3) that integrates genomic annotations (Probeset Data in Tabular Format) from the NetAffx™ Analysis Center [11], single probe mappings over NCBI RefSeqs and EMBL cDNAs generated at ISREC using the software *tagger *[12] and the exon-intron genomic coordinates of each known human transcript downloaded with the UCSC Table Browser [13]. The tables mapping single probe pairs to RefSeq and cDNAs are part of the CleanEx database [14]. The integrated annotations are stored into a MySQL[15] database; it is possible to automatically update a set of tables related to GeneChip^® ^platform with the perl script *add_chip.pl*. Two Object Oriented Perl modules are the core of the system: Splicy::AffyDB and Splicy::Probeset.

The first module parses raw data in tabular and comma-separated format, and inserts this data into a set of MySQL tables. The second module is used to store probeset data into a Perl object at runtime and to manipulate this data in order to generate the graphical displays. Splicy currently runs on a public web-server at the IFOM-IEO research institute [1].

The program accepts in input a GeneChip^® ^platform, a class of putative targets (RefSeq, cDNAs or both) and a list of objects to be queried. A query object can be a *Probeset ID*, a *RefSeq accession*, a *Gene Symbol *or an *Affymetrix*^® ^*Representative Public ID*. A Representative Public ID is a sequence (chosen during chip design) which is optimally associated with the transcribed region that is interrogated by the probeset [11]. Once the given object (transcript or probeset) is identified, Splicy organizes the informations by Probeset ID and produces a series of graphical displays showing the association between the probeset and the transcripts targeted by the probes.

For each Probeset retrieved, Splicy can display a graphical report (Probe Maps) or can generate two different tsv (tab-separated) files: one containing the information related to the Probeset and another focused on the Probe Pair data.

We currently maintain mappings of the most popular human and mouse Affymetrix™ GeneChips^®^, and Splicy can be queried for matches with human and mouse RefSeqs and EMBL cDNAs.

### Probe Maps and 'splice diagnostic probes'

**Figure 4:**
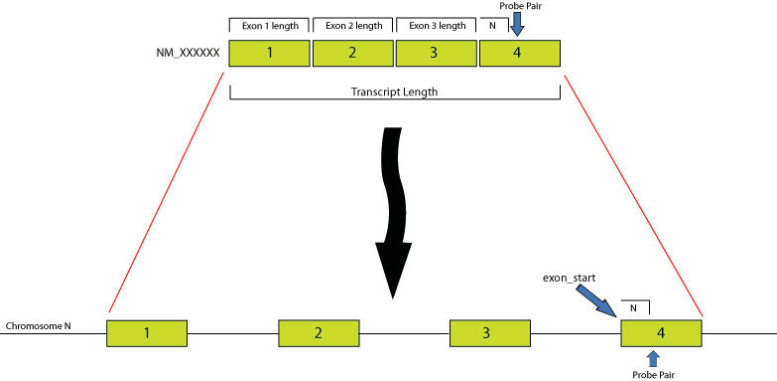
Projection of coordinates relatives to the transcript structure over the genome. Once the intron and exon positions on the chromosome are collected, Splicy first finds in which exon the single probe matches, then projects this information on the chromosome coordinates.

Each graphical display is generated from numeric data using the GD graphic library [16] and the Perl module Bio::Graphics, part of the BioPerl distribution [17]. Splicy maintains static coordinates data relative to alignments between probes and transcripts (start and end of probes alignments and length of the transcripts). At runtime the module Splicy::Probeset.pm uses the intron-exon genomic coordinates to convert *transcript-relative *coordinates to genomic coordinates (Figure 4).

**Figure 5:**
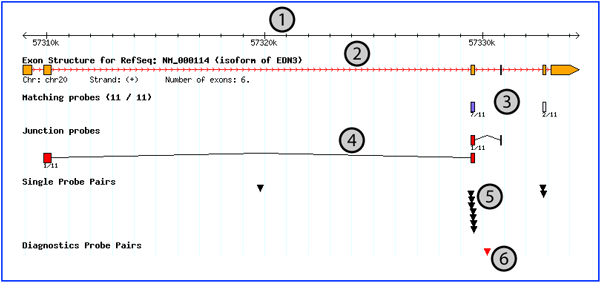
A graphical report generated with the BioPerl library Bio::Graphics stores the coordinates on the chromosome (1), the intron-exon structure of the transcript (2), the probes matching on specific exons (3), the Junction probes (4), triangular glyphs representing single probes (5) and *splice diagnostic probes *(6).

Each image (Figure 5) reports a line showing the position of the transcript on the chromosome (5.1) and the genomic exon-intron structure for a transcript associated with the probes (5.2). Below the transcript structure there are some glyphs (boxes) used to highlight the exons containing one or more matching probes; these boxes are marked with the number of probe pairs matching with this specific exon (5.3). A second line of red boxes connected by segments (5.4) underline "*Junction Probes*" which are at the boundary of two exons. These probes are particularly interesting because if one of the two exons involved in the hybridization is skipped in an alternative isoform, the probes can produce different hybridization patterns. The last lines in the graphical display show, as triangular glyphs, the position of single probes over the transcript (5.5). If a given probe belongs to an exon which is skipped in a different transcript (isoform) of the same gene, it is tagged as a potential 'splice diagnostic probe' and marked red (5.6). The idea is that a given probeset containing 'splicing diagnostic probes' will behave differently in the hybridization process, according to the transcript variant present in the hybridization mixture. The images are associated with HTML client-side maps that associate the triangular glyphs (position of single probes) with a pop-up generated with the Javascript library Overlib [18]: when the user mouse is over a specific glyph, Splicy generates a small pop-up showing: probeset ID, oligonucleotide sequence, X and Y on the array, position of the alignments on the given target transcript ([start stop]length_of_the_transcript). All the annotation data stored statically on the server can be retrieved from the graphical interface using a set of buttons on the top of the graphic report: Design (description of the Representative public ID), Targets (all the transcripts matching with the selected probeset), Probe Pairs (nucleotide sequence and position on the array of the single probes), Alignments (coordinates of the genome alignments), Notes and Links (further notes and links related to the target representative public ID), Function (GO functional classification). Splicy provides also a direct link to the Entrez Gene [19] entry corresponding to the target gene and (at the bottom of the record) direct links to the UCSC genome browser [20].

### Tab-separated files

The tab-separated files contain annotations and the mapping data related to the transcripts and/or chromosomes, starting from a given list of objects (*Probeset ID, Gene Symbols, Representative Public ID, RefSeq*).

Probeset and Probepairs informations are available for download; the user can interactively select which kind of data to include in the output. Two different files will be generated, one containing information related to the Probeset ID (file suffix PS_) and another file containing information related to single probe pairs (file suffix PP_). Once the user has selected which kind of data to include in the output, the files are generated into a temporary directory accessible by the user. If a user selects more than 30 objects, an e-mail address is requested, and the server sends an e-mail to the user once the requested file is complete.

### GUI Interface

The Splicy interface is flexible and user-friendly. The first page contains links to the following sections: Probe Maps, TAB files, statistics, help, source code and documentation.

The Probes Maps form allows the user to select a GeneChip^® ^platform with a set of target transcripts (human and mouse RefSeq and EMBL cDNAs) and to insert a list of query objects (*Probeset ID*, *RefSeq accession*, *Gene Symbols*, *Representative Public ID*).

The TAB Files form is composed by three windows: General Info allows the user to select a GeneChip^® ^platform, a set of target transcripts, a list of query objects and an e-mail address; the Probeset frame allows the selection of data related to the probeset (GeneChip^® ^informations, sequence design informations, RefSeq targets, Alignments, Functional GO annotation); the Probe Pairs window enables selection of data related to the single probes (position on the array, sequence, probe mapping on the genome and on the target transcripts). The statistics page contains general information about the number of platforms available in the server (number of GeneChips^® ^available, number of probesets, number of splice diagnostics probesets for RefSeqs targets and for EMBL cDNAs targets). Help and Documentation pages describe the use of the Web-Inteface and of the Perl modules.

## Availability and requirements

• **Project Name: **Splicy – GeneChip^® ^Splice Machine

• **Project HomePage: **[1]

• **Operating System: **Splicy is currently running on a FreeBSD 5.4 server

• **Programming Language: **Perl, MySQL, Javascript

• **Other requirements: **Apache 2.0, MySQL 4.0 or higher, Perl 5.8

• **License: **the Splicy server is freely available on the web [1]. Researchers are not required to pay for access or data download. The Splicy package and the Splicy Perl modules (AffyDB.pm and Splicy.pm) are under GNU-GPL License.

## Authors' contributions

DR was responsible for the program design and development. AG conceived and supported the project. BF produced the compilation of available isoforms. DC contribute to the software development and deployed the software on a FreeBSD Server. VP and DB are the driving force behind the CleanEx Expression Reference Database Project, which is the basis for the probeset-exon mapping.
